# 3V-GM: A Tri-Layer “Point–Line–Plane” Critical Node Identification Algorithm for New Power Systems

**DOI:** 10.3390/e27090937

**Published:** 2025-09-07

**Authors:** Yuzhuo Dai, Min Zhao, Gengchen Zhang, Tianze Zhao

**Affiliations:** Beijing Key Laboratory of Network System Architecture and Convergence, Beijing University of Posts and Telecommunications, Beijing 100876, China; dyz@bupt.edu.cn (Y.D.); buptzgc@bupt.edu.cn (G.Z.); zhaotz@bupt.edu.cn (T.Z.)

**Keywords:** new power system, critical node, three-dimensional gravity model, topological and electrical feature fusion, MATPOWER

## Abstract

With the increasing penetration of renewable energy, the stochastic and intermittent nature of its generation increases operational uncertainty and vulnerability, posing significant challenges for grid stability. However, traditional algorithms typically identify critical nodes by focusing solely on the network topology or power flow, or by combining the two, which leads to the inaccurate and incomplete identification of essential nodes. To address this, we propose the Three-Dimensional Value-Based Gravity Model (3V-GM), which integrates structural and electrical–physical attributes across three layers. In the plane layer, we combine each node’s global topological position with its real-time supply–demand voltage state. In the line layer, we introduce an electrical coupling distance to quantify the strength of electromagnetic interactions between nodes. In the point layer, we apply eigenvector centrality to detect latent hub nodes whose influence is not immediately apparent. The performance of our proposed method was evaluated by examining the change in the load loss rate as nodes were sequentially removed. To assess the effectiveness of the 3V-GM approach, simulations were conducted on the IEEE 39 system, as well as six other benchmark networks. The simulations were performed using Python scripts, with operational parameters such as bus voltages, active and reactive power flows, and branch impedances obtained from standard test cases provided by MATPOWER v7.1. The results consistently show that removing the same number of nodes identified by 3V-GM leads to a greater load loss compared to the six baseline methods. This demonstrates the superior accuracy and stability of our approach. Additionally, an ablation experiment, which decomposed and recombined the three layers, further highlights the unique contribution of each component to the overall performance.

## 1. Introduction

With the rapid advancement of new power systems, renewable generation now occupies an ever-growing share of the energy mix. Renewable sources, such as wind and solar, are inherently stochastic, intermittent, and volatile, which substantially increases the uncertainty of generation and complicates grid maintenance and operational procedures. Consequently, traditional operating paradigms and security assurance mechanisms face entirely new challenges [1].

Due to the inherent characteristics of renewable generation, new power systems face greater uncertainty and risk. When sudden events occur, such as natural disasters, malicious cyberattacks, or equipment failures caused by aging infrastructure, power control systems and other critical assets may suffer severe damage, resulting in large-scale blackouts [2]. These outages constitute major system incidents that result not only in extensive load shedding but also in serious social and economic repercussions [3].

On 28 April 2025, a large-scale blackout occurred in Spain and Portugal, affecting millions of residents, paralyzing public services, and disrupting social order. This incident once again underscores the critical importance of power grid stability and highlights the essential role of identifying key nodes within power systems. Historically, numerous incidents have demonstrated the severe consequences of failures at critical nodes. For example, in 2016, an extreme weather event in Australia caused a large-scale blackout due to the disconnection of renewable energy sources. In August 2019, the unexpected disconnection of two wind farms caused the frequency of the UK’s national grid to fall outside its normal operating range, leading to a nationwide power outage. In April 2019, Venezuela experienced two consecutive nationwide blackouts due to combined physical and cyberattacks on its power system. These events collectively highlight the growing importance of accurately identifying critical nodes in power systems and implementing targeted protection measures to improve system security and stability.

Therefore, enhancing the stability and robustness of power systems has become a key research focus in the field of power engineering. The identification and protection of critical nodes, as structural defense measures, are widely recognized as effective strategies to ensure secure system operation. A critical node is defined as one whose failure can significantly affect the overall functionality and stability of the system. Studies have shown that failures of these nodes often precipitate large-scale blackouts [4]. Consequently, the precise identification and targeted protection of critical nodes are crucial in enhancing grid resilience and mitigating systemic risks.

Currently, critical node identification methods can be broadly classified into two main categories. The first consists of traditional topology-based approaches, which identify nodes that significantly impact network functionality and stability using global or local metrics. Classic algorithms in this category include degree centrality [5], betweenness centrality [6], clustering coefficients [7], the K-shell algorithm [8], structural holes [9], and PageRank [10].

The second category covers a range of innovative algorithms built on these classical foundations. For example, reference [11] enhances K-shell decomposition by incorporating structural hole principles and accounting for first- and second-order neighbors, resulting in the SHKS ranking algorithm. In [12,13], the critical nodes are evaluated using gravity models. Reference [14] leverages an improved effective distance fusion model to identify important nodes (IEDG), which combines the node degree, K-shell value, and eigenvector centrality to characterize positional, local, and global influences jointly. Building on local structural characteristics, reference [15] introduced the LWC algorithm and applied an entropy weight method to fuse multiple local metrics into a single comprehensive ranking. Finally, reference [16] presented an improved TOPSIS approach that leverages node communication probabilities and information entropy to identify key nodes from global, local, and positional perspectives. These advances have markedly improved both the accuracy and practical applicability of critical node identification.

However, with the ongoing expansion of power grids and the growing demand for and uncertainty of generation associated with the integration of renewable energy, traditional topology-based approaches are revealing their limitations. Existing vulnerability analysis methods often rely solely on topological characteristics, overlooking the crucial role of power flow information in determining the operational capacity and stability of the grid.

To address these shortcomings, recent research has focused increasingly on integrating electrical operational characteristics with structural information in the identification of critical nodes. For example, references [17,18,19] combine power flow data with topological metrics, employing the weighted fusion of multiple local importance scores to assess the significance of the nodes. Reference [20] further incorporates power information at the node level, evaluating the importance of the node by jointly considering the importance and dependence of power supplies and loads. While these approaches have improved the joint consideration of electrical and topological features, they still fall short in fully capturing the complex internode relationships and global coupling characteristics inherent in power networks, limiting their practical applicability in real-world scenarios.

This paper conducts an in-depth analysis of the multiple influencing factors affecting node criticality in power systems, revealing that the importance of a node is not only determined by its topological position and power flow characteristics but requires a comprehensive evaluation across multiple interrelated dimensions. On this basis, a critical node identification algorithm is proposed on the basis of the concept of a minimum resistance surface. The algorithm integrates the topological structure of the power network, the distribution characteristics of the power flow, and the electrical coupling distances between the nodes, allowing the accurate identification of critical nodes that play a vital role in the stability and secure operation of the network.

This paper contributes as follows.

1. Construction of 3V-GM: We introduce an innovative three-dimensional critical node identification algorithm, namely 3V-GM, which synergizes the network topology with the power flow distribution. In the plane layer, the minimum-resistance surface model is used to capture the global position and supply–demand coupling status of each node. In the line layer, the electrical coupling distance between nodes is quantified to characterize the tightness and coordination of the power transmission paths. In the point layer, eigenvector centrality is employed to identify latent hub nodes with global influence that are not immediately evident from the network structure. The 3V-GM algorithm surpasses the limitations of traditional algorithms that rely on single-indicator evaluation or simple weighted combinations, enabling a multi-scale and in-depth, critical analysis that spans global structures to local mechanisms and macroscopic topologies to microscopic behaviors.
2. Introduction of PIMRS: At the plane layer, our study creatively introduces the expansion resistance factor to the evaluation of critical nodes, which couples supply–demand imbalance with voltage deviation to construct a minimum-resistance surface model with clear physical significance, namely the Physically Inspired Minimum-Resistance Surface Model (PIMRS). PIMRS integrates K-shell hierarchical features with degree centrality, effectively addressing the limitation of the traditional K-shell algorithm in capturing differences among nodes within the same shell. As a result, it enhances the model’s ability to distinguish and accurately locate high-risk nodes under real-world power grid conditions.
3. Development of LRF simulation mechanism: To more accurately reflect the impact of critical node failures on power system operation, we design an internal load redistribution mechanism that simulates the dynamic scheduling behavior of the system following faults, namely the load redistribution fault (LRF) simulation mechanism based on scheduling behavior. By incorporating this scheduling and control process into the load loss assessment, the model more closely approximates real-world operational responses, thereby enhancing the accuracy of resilience and vulnerability assessments for nodes in power grids.

## 2. Preliminaries

The topology of a power system can be represented as a network of nodes and edges, where each node denotes a functional unit in the grid, such as power plants (source nodes), substations or relay stations (relay nodes), and electrical equipment (load nodes). The edges describe the energy or power transmission relationships between these nodes. Based on graph theory, the topological structure of the power communication network is defined as G(V,E), where $V=\{v_{1},v_{2},\ldots ,v_{N}\}$ represents the set of nodes with a total number of |V|=N, and $E=\{e_{1},e_{2},\ldots ,e_{K}\}$ is the set of undirected edges with a total number of |E|=K. The adjacency matrix of *G* is defined as $A={\left\{a_{ij}\right\}}_{N\times N}$; if there are connected edges between node *i* and node *j*, then $a_{ij}=1$; otherwise, $a_{ij}=0$.

### 2.1. Degree Centrality [5]

The degree of a node represents the number of direct connections that it has with other nodes. A higher degree implies that the node is more strongly integrated within the network structure and potentially exerts a greater influence. Degree centrality is a normalized metric that quantifies this connectivity. The degree centrality of node *i* is defined as

(1) $$DC_{i}=\sum_{j=1}^{N}\frac{a_{ij}}{N-1}$$

where *N* represents the number of nodes in the network.

### 2.2. Betweenness Centrality [6]

Betweenness centrality is a global network metric that represents the probability that communication between any two nodes in the network passes through the given node. Higher betweenness centrality indicates a greater likelihood that the node functions as a bridge for information flow, thereby highlighting its strategic importance in facilitating communication across the network. The betweenness centrality of node *i* is defined as

(2) $$BC_{i}=\frac{2}{N\left(N-1\right)}\sum_{j\neq k\neq i\in V}\frac{g_{jk}\left(i\right)}{g_{jk}}$$

where $g_{jk}$ represents the number of shortest paths between any two nodes *j* and *k*, while $g_{jk}\left(i\right)$ denotes the number of shortest paths between nodes *j* and *k* that pass through node *i*.

### 2.3. K-Shell Algorithm [8]

The K-shell algorithm is a method of hierarchically partitioning the nodes in a network based on their degrees. The specific steps are as follows.

1. Step 1: Identify all nodes in the network with a degree of 1 and then remove these nodes and their connected edges. Continue this process in the remaining network by repeatedly identifying and removing nodes with a degree of 1. Once no nodes with a degree of 1 remain, the removed nodes form the first layer, with K=1.
2. Step 2: Identify all nodes in the network with a degree of 2 and then remove these nodes and their connected edges. Continue this process by identifying and eliminating nodes with a degree of 2 or less in the remaining network. Once such nodes are removed, the removed nodes form the second layer, with K=2.
3. Step 3: Repeat Step 2 iteratively, progressively removing nodes with higher degrees until all nodes in the network are removed and assigned a corresponding *K* value.

### 2.4. Gravity Model Algorithm Based on K-Shell (KSGC) [13]

The gravity-based critical node algorithm uses the degree of a node as its mass in the gravity model and the number of edges on the shortest path between two nodes as the distance between them. Building on this foundation, the KSGC method further incorporates the structural characteristics of complex networks. This approach posits that nodes located in the network’s core region exhibit higher stability compared to those on the periphery. The rationale is that core nodes are more likely to receive support from other nodes in the event of an attack or failure. Consequently, central nodes possess a stronger “gravitational pull” or attractive influence than peripheral ones.

To more accurately capture this structural impact, the KSGC method introduces hierarchical information derived from K-shell decomposition when computing the interaction between nodes. In addition to considering the node degree and shortest path distance, it incorporates the K-shell layer difference between two nodes as a measure of positional disparity. Based on this, the attraction coefficient from node *i* to node *j* is defined as follows:

(3) $$C_{ij}=e^{\frac{K_{i}-K_{j}}{K_{\max}-K_{\min}}}$$

where $K_{i}$ represents the K-shell value of node *i*; $K_{\max}$ and $K_{\min}$ are the maximum K-shell and minimum K-shell values in the network.

By simultaneously considering the network truncation radius, the importance of node *i* based on the gravity model, denoted as $KSGC_{i}$, is defined as follows:

(4) $$KSGC_{i}=\sum_{d_{ij}\leq \frac{1}{2}d_{\max}}C_{ij}\cdot \frac{D_{i}\cdot D_{j}}{d_{ij}}$$

where $D_{i}$ and $D_{j}$ represent the degrees of node *i* and *j*, respectively. $d_{ij}$ represents the shortest distance between nodes *i* and *j*. $d_{\max}$ indicates the maximum network path length.

The KSGC algorithm integrates the attributes of local nodes and the global network structure by introducing an attraction coefficient derived from K-shell decomposition. Locally, it accounts for the degree of the node and the shortest path distances to assess its connectivity within its neighborhood. Globally, it incorporates the differences in the K-shell layers to capture the structural centrality of the node within the entire network. The resulting KSGC score reflects both the local influence and global prominence of a node. A higher KSGC score indicates a more critical role of the node in maintaining network stability and facilitating information dissemination.

## 3. Materials and Methods

**Figure 1:**
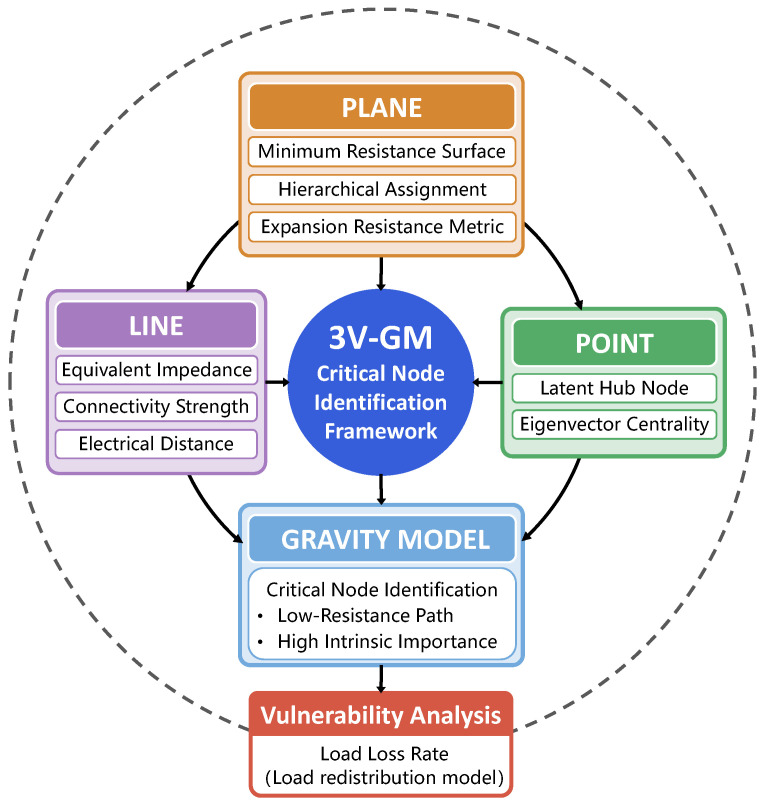
Workflow schematic of the 3V-GM framework.

We propose a multi-dimensional critical node identification framework for power networks, namely 3V-GM, which integrates “point–line–plane” features, as illustrated in Figure 1. In the plane layer, a minimum-resistance surface model is constructed by introducing a resistance factor and combining K-shell values with degree centrality, enabling a comprehensive assessment of node importance based on both the global topological centrality and local connectivity density. In the line layer, an electrical coupling distance is introduced to quantitatively describe the impedance characteristics of power transmission paths, revealing potential cascading failure channels. In the point layer, eigenvector centrality is used to identify latent hub nodes with global influence that are often overlooked by traditional centrality measures. Based on the multi-dimensional information, we further develop a gravity model-based evaluation system to achieve the unified integration of the extracted features. The resulting method effectively identifies critical nodes with high potential energy located along low-impedance paths, offering strong theoretical support for power grid stability assessment and control strategy optimization.

The operating parameters used in all methods in this study, such as bus voltages, active and reactive power flows, and branch impedances, were directly obtained from standard test cases provided by MATPOWER v7.1. Topological metrics, including degree centrality and K-shell values, were derived from the adjacency matrices of these test networks. Power flow analysis and impedance matrix construction were performed using MATPOWER, while custom Python scripts were employed for multi-dimensional feature extraction and the evaluation of the gravitational model.

### 3.1. Plane Layer: Global Minimum-Resistance Surface

In plane-layer analysis, the minimum cumulative resistance model constructs a multi-layered resistance surface to more accurately characterize the global position of each node within the network and its mutual influence on others. The effectiveness of this model depends on the scientific formulation of the resistance surface, which comprises two core components: the hierarchical assignment and the expansion resistance metric.

Regarding hierarchical assignment, the traditional K-shell decomposition method accurately reflects the hierarchical positions of nodes within the network. However, it suffers from a critical limitation: nodes within the same K-shell layer are assigned identical evaluation values. This uniform treatment neglects the heterogeneity among nodes within the same layer. In a real network, nodes with the same K-shell value may differ significantly in importance due to variations in their interlayer connections.

**Figure 2:**
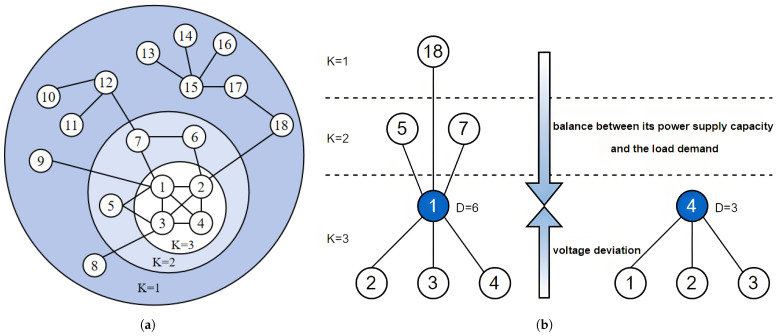
Structural hierarchy and expansion resistance-based evaluation in the plane layer. (**a**) K-shell decomposition diagram. (**b**) Illustration of the improved plane-layer evaluation. Although nodes 1 and 4 belong to the same K-shell layer, they differ in degree, structural influence, power supply–demand characteristics, and voltage deviation, resulting in significant differences in importance. Therefore, the expansion resistance factor is introduced to provide a more comprehensive evaluation of each node’s operational stress and criticality.

In Figure 2a, although nodes 1 and 4 are positioned at the same level (K = 3), their roles differ significantly. Node 1 demonstrates strong control and intermediary capacity by maintaining direct connections with nodes at different levels, which resembles the rich club phenomenon. The rich club phenomenon suggests that the core nodes in a network tend to establish denser connections with other core nodes, while having fewer connections to peripheral nodes [21]. This leads to a decrease in the efficiency of information dissemination. As a result, node 1 can effectively propagate information across layers, enhancing its influence within the network. In contrast, node 4 is only connected to peers at the same level, which limits its propagation range and information flow capacity.

To overcome the limitations of traditional methods, we introduce an innovative expansion resistance factor *R* to quantify the compound operational pressure experienced by a node in terms of voltage stability and power supply–demand balance, as illustrated in Figure 2b. The expansion resistance factor of node *i* is defined as follows:

(5) $$R_{i}=\left|S_{\mathrm{gen},i}-S_{\mathrm{load},i}\right|\cdot \left|U_{i}-U_{\mathrm{nom}}\right|,$$

(6) $$S_{\mathrm{gen},i}=\sqrt{P_{\mathrm{gen},i}^{2}+Q_{\mathrm{gen},i}^{2}},$$

(7) $$S_{\mathrm{load},i}=\sqrt{P_{\mathrm{load},i}^{2}+Q_{\mathrm{load},i}^{2}}$$

where $S_{\mathrm{gen},i}$ and $S_{\mathrm{load},i}$ represent the apparent power generation and load at node *i*, respectively. $P_{\mathrm{gen},i}$ and $Q_{\mathrm{gen},i}$ denote the active and reactive power generation at node *i*, respectively, while $P_{\mathrm{load},i}$ and $Q_{\mathrm{load},i}$ denote the active and reactive power load at node *i*. $U_{i}$ is the voltage magnitude at node *i*, and $U_{\mathrm{nom}}$ is the nominal voltage of the system, usually set at 1 pu, as is standard in the analysis of power systems.

The expansion resistance factor fully captures the balance between the power supply capacity of a node and the load demand. It also incorporates voltage deviation as a key indicator of the node’s deviation from steady-state operation, forming a compound feature that couples supply–demand balance and voltage stability. This factor reflects the critical notion that, when a node exhibits a significant power imbalance and its voltage substantially deviates from nominal levels, it is highly likely to become a structural weak point in system operation [22]. This type of node tends to be more sensitive to disturbances. It has higher potential for fault propagation, making it a probable trigger for local instability or even cascading failures at the system level.

Incorporating the expansion resistance factor into the weighting mechanism of the minimum-resistance surface effectively overcomes the limitations of the traditional K-shell method, which assigns uniform importance to nodes within the same layer. It significantly enhances the model’s ability to express internal heterogeneity among nodes and improves its discriminative performance in real-world operational scenarios. As a key component at the plane layer, the expansion resistance factor is further embedded in the gravity model framework, enabling deep coupling and interactive analysis between the network topology and the physical electrical state of the system.

Algorithm 1 presents the pseudocode for calculating the expansion resistance factor. In a power system, a node may be connected to multiple generators, and, to assess the total generation capacity (active and reactive power) of each node, the output power of all generators at that node must be summed. The expansion resistance factor combines both active and reactive power to characterize the importance of each node in the network comprehensively. Active power indicates the load-bearing capacity of a node along transmission paths, serving as a key metric for its central function and the potential impact of faults. Reactive power quantifies a node’s ability to regulate voltage stability, as reactive fluctuations directly impact the voltage stability margin and the risk of instability. By the vector synthesis of these two components, the model achieves a unified and dimensionally consistent representation of the actual energy interaction intensity of a node.
**Algorithm 1:** The Expansion Resistance Factor Calculation
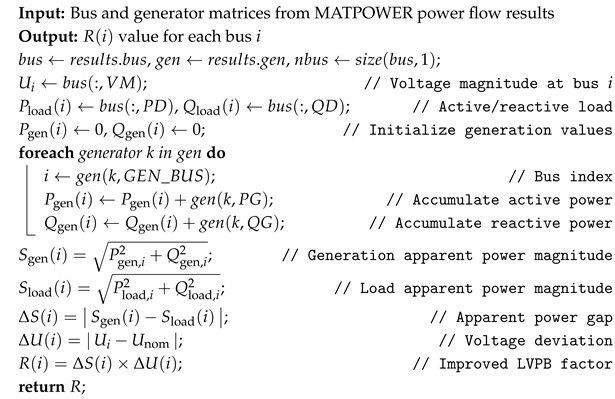


In power networks, the plane-layer importance of a node is defined through a comprehensive evaluation of its position in the global topology and its electrical characteristics. To accurately measure node importance, we introduce degree centrality and the K-shell value, while adjusting the node’s electrical characteristics using the expanded resistance factor. Specifically, the plane-layer importance of node *i* is defined as follows:

(8) $$I_{i}=e^{-R_{i}}\left(\frac{D_{i}}{D_{\max}}+\frac{K_{i}}{K_{\max}}\right)$$

where $R_{i}$ is the expansion resistance factor. $D_{i}$ denotes the degree of node *i*, and $D_{\max}$ is the maximum degree of node *i*. $K_{i}$ represents the K-shell value of node *i*, and $K_{\max}$ is the maximum K-shell value of node *i*.

The introduction of the expanded resistance factor $R_{i}$ is a crucial aspect of this study. It evaluates the electrical pressure on a node during power transmission, focusing on voltage stability and the balance between power supply and demand, thus overcoming the limitations of traditional topological analysis methods. Unlike the conventional K-shell method, which focuses solely on node connectivity, the expanded resistance factor is based on electrical principles. This approach enables the identification of nodes that may not be prominent in the topological structure but carry higher electrical loads. These nodes can become weak links in the system due to voltage fluctuations or imbalances between supply and demand, making them potential sources of fault propagation.

Therefore, the definition of the plane-layer importance is based not only on the topological positions of nodes but also on their operational states in the power system. By combining topological features with electrical characteristics, this method effectively identifies high-potential nodes located along low-resistance paths in the power network. These nodes play a critical role in the system’s operation and are also key points for fault propagation. This approach provides a more comprehensive and precise theoretical basis for stability analysis, fault prediction, and optimization scheduling in power networks.

### 3.2. Line Layer: Electrical Coupling Distance Between Nodes

In the line layer, 3V-GM focuses on the electrical connectivity characteristics between the nodes in the power network. To precisely capture the coupling relationships, we introduce the concept of the electrical coupling distance, which is computed as the equivalent impedance between two nodes based on the node impedance matrix. This metric quantitatively reflects the strength of electromagnetic coupling from a physical perspective. A smaller electrical coupling distance indicates a stronger electrical link between the nodes, making them more likely to form power interaction channels and disturbance propagation paths during system operation. As a result, such connections increase the risk of voltage fluctuations, phase angle deviations, and even cascading failures.

**Figure 3:**
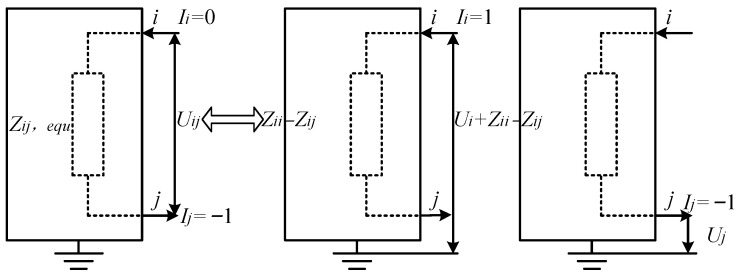
Computing the electrical distance between buses in a power grid [23].

The electric distance between node *i* and *j* in a power network can be defined as the equivalent impedance $Z_{ij,\mathrm{equ}}$. Numerically, $Z_{ij,\mathrm{equ}}$ is equal to the voltage difference $U_{ij}$ between node *i* and *j* when a unit current is injected into node *i*:

(9) $$Z_{ij,\mathrm{equ}}=\frac{U_{ij}}{I_{i}}=U_{ij}$$

where $U_{ij}$ represents the voltage between node *i* and node *j*, and $I_{i}$ represents the current of node *i*, as shown in Figure 3.

Based on the superposition principle and reference [24], the elements of the system nodal impedance matrix can be defined as follows:

(10) $$Z_{ij,\mathrm{equ}}=\left(Z_{ii}-Z_{ij}\right)-\left(Z_{ij}-Z_{jj}\right)$$

where $Z_{ij}$ represents the impedance between node *i* and node *j*.

The electrical distance between nodes is a key metric in quantifying the strength of interactions in power networks. It precisely characterizes the tightness of electrical connectivity by measuring the equivalent impedance between pairs of nodes. A smaller electrical distance indicates stronger electrical coupling, lower impedance along power transmission paths, and a more substantial mutual influence on the voltage and phase angle.

This metric reflects not only the topological connectivity within the network but also functional interdependencies from the perspective of electromagnetic coupling. In power system operation, the electrical distance directly influences dynamic interactions between nodes, with nodes in closer electrical proximity being more vulnerable to cascading effects, such as voltage fluctuations and phase angle instability.

### 3.3. Point Layer: Identification of Latent Hub Nodes

In the point layer, we introduce the eigenvector centrality to capture the global latent influence of the nodes. It calculates the principal eigenvector of the network topological adjacency matrix, assessing not only the importance of a node but also the influence of its neighboring nodes. As a result, it enables the identification of hidden hub nodes that are often overlooked by traditional centrality measures.

Such nodes are typically located at the intersections of multiple critical power transmission paths. Their failures are highly coupled with other network components, making them particularly prone to triggering large-scale blackout events that exceed expected failure limits. The formula for calculating the eigenvector centrality is defined as follows:

(11) AX=λX,

(12) $$e_{i}=X_{i}$$

where λ and *X* represent the largest eigenvalue and the corresponding normalized eigenvector, respectively. $e_{i}=X_{i}$ denotes the eigenvector component associated with node *i*, reflecting its global influence throughout the network.

Based on the above analysis, a fused importance score $I_{i}$ is introduced and combined with the eigenvector component $e_{i}$ through weighted integration [25]. The composite importance index $V_{i}$ for each node is defined as follows:

(13) $$V_{i}=e_{i}\cdot I_{i}.$$

This indicator enables an integrated evaluation of structural centrality and physical operational characteristics, providing a theoretical foundation for the precise identification of latent global critical nodes.

### 3.4. Improved Gravity Model

Building on the above foundation, we introduce a gravity model to construct a unified evaluation algorithm to identify critical nodes. In this approach, the power network is analogized to a dynamic potential field, where nodes are treated as entities that possess both “mass” (composite influence) and “gravitational force” (controlling power). The strength of the interaction between the nodes corresponds to their potential to influence the system.

By simulating a mechanism similar to Newton’s law of gravitation, the model quantifies the internode forces in three dimensions: the structural position, operational status, and physical coupling characteristics. This allows for the identification of potential hub nodes that exert significant control and influence within the network [26]. Consequently, the influence of the node *i* is defined as follows:

(14) $${3\text{V-GM}}_{i}=\sum_{\begin{array}{c} j\neq i\\ j\in V \end{array}}\frac{V_{i}V_{j}}{Z_{ij,\mathrm{equ}}^{2}}.$$

**Figure 4:**
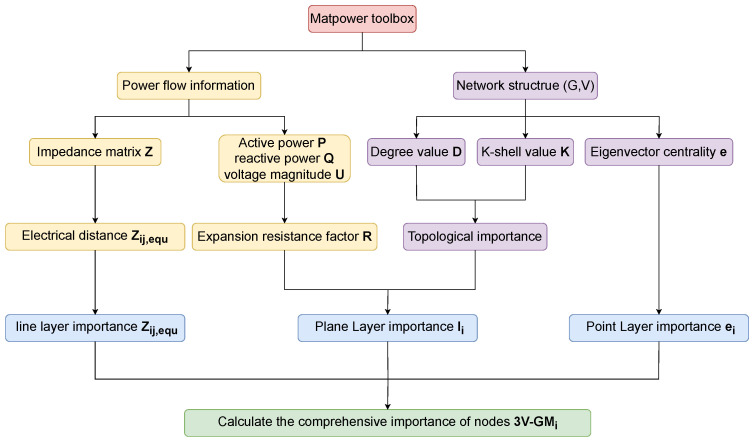
The algorithm framework flow of 3V-GM.

Traditional gravity model approaches for the identification of critical nodes in power grids define “mass” solely based on topological metrics, such as degree centrality, K-shell values, or structural holes. They measure “distance” using the shortest or alternative paths within the network topology. In contrast, our proposed 3V-GM framework represents significant advancements in both areas. First, it combines each node’s operational state with its topological importance, giving “mass” both operational and structural significance. Second, it replaces static topology-based distances with a dynamic electrical coupling distance, which accurately quantifies the strength of electromagnetic interactions between nodes. Through this algorithm, it becomes possible to effectively identify critical nodes that have a high composite influence and are located along low-impedance transmission paths. These nodes often serve as core control points for network stability or as potential sources of fault propagation. Their identification is of great significance in improving grid resilience and optimizing system dispatch strategies. The 3V-GM method proposed in this paper can be implemented following the workflow shown in Figure 4.

## 4. Vulnerability Analysis

In complex network theory, the process of analyzing network performance by removing network elements is referred to as network vulnerability analysis. The fundamental function of a power system is to provide enough electricity to charge the nodes. The load loss rate measures the proportion of unmet load caused by node or line failures in a given fault scenario relative to the total demand. This metric is instrumental in evaluating the resilience and reliability of the power grid in the face of attacks or operational failures [27]. The network loss load rate is defined as follows:

(15) $$LR=\frac{Loss}{L}$$

where Loss represents the load lost in the network after the nodes are attacked, and *L* denotes the total load of the network in its initial state.

In modern power systems, cascading failure control models are commonly employed to enhance grid robustness and resilience under fault conditions. We use a node failure-based load redistribution model, where the failure of a node triggers the central control system to promptly redistribute its load to neighboring nodes in a balanced and controlled manner. Any portion of the load that exceeds the carrying capacity of adjacent nodes is discarded and recorded as a load loss. This process is carried out exclusively for load nodes; source nodes bear no additional load and participate in the redistribution calculations. This mechanism effectively reduces the rate of load loss resulting from individual node failures, thereby improving the overall stability and reliability of the power grid. In addition, it helps to prevent large-scale blackouts caused by cascading effects.

The maximum load capacity of a node refers to the highest load that it can support, mainly influenced by construction costs. Generally, the maximum capacity of node *i* is positively correlated with its initial load $F_{i}$ and can be expressed as [28]

(16) $$C_{i}^{\max}=\left(1+\alpha \right)F_{i},i=1,2,\ldots ,N$$

where α is the capacity parameter of nodes, which is a constant for a given network.

In a network, when a node fails, its load is redistributed to other load nodes according to an allocation method. At the start of the algorithm, the system classifies each neighbor of a failed node as a "source node" or a "load node". During load redistribution, only load nodes receive redistributed loads. Because the source nodes have an initial load of zero and, consequently, a maximum load capacity of zero, they cannot support additional loads. Therefore, in redistribution calculations, further distinguishing node types is unnecessary: if a source node is encountered, any load assigned to it is considered lost and is immediately discarded. During the redistribution process, the preferential allocation principle is followed [29], and the probability of redistributing the load from the failed node *i* to a neighboring node *j* is given by

(17) $$\tau_{j}\;=\;\frac{k_{j}^{\beta }}{\sum_{m\in \Gamma_{i}}k_{m}^{\beta }}$$

where $k_{i}^{\beta }$ represents the degree of node *i* with an adjustable load parameter β (β > 0), *j* represents a neighboring node of the failed node *i*, and $\Gamma_{i}$ denotes the set of neighboring nodes of node *i*. Thus, the additional load assigned to the neighboring node *j* is

(18) $$\Delta F_{j}=F_{i}\times \tau_{j}=F_{i}\;\frac{k_{j}^{\beta }}{\sum_{m\in \Gamma_{i}}k_{m}^{\beta }}\;.$$

Case 1: If node *j*, after receiving the additional load $\Delta F_{j}$, has a total load $F_{j}+\Delta F_{j}$ that does not exceed its capacity limit $C_{j}$, the node can safely accommodate the additional load. Thus, the final load of node *j* is given by the following formula:

(19) $$F_{j}=F_{j}+\Delta F_{j}.$$

Case 2: However, if the additional load received by node *j* causes its total load to exceed its capacity $C_{j}$, the node can only accept a portion of the load up to its maximum capacity $C_{j}$, resulting in a load loss. The calculation is as follows:

(20) $$Loss=\sum_{j\in I}F_{j}+\Delta F_{j}-C_{j}$$

where *I* represents the set of all nodes that exceed their capacity limits, and the load of node *j* is updated to its maximum capacity as follows:

(21) $$F_{j}=C_{j}.$$

Therefore, when a node in the network fails, its load loss is the total sum of the loads that its neighboring nodes cannot accommodate. As the nodes are individually attacked, the network load loss gradually accumulates until the load loss rate reaches 100%.

## 5. Results

All algorithms were simulated and validated within Python 3.8 using TensorFlow 1.13/2.0. The computations were executed on a standard PC platform equipped with an Intel Core i5 processor, 8 GB RAM, and integrated graphics. No dedicated GPU acceleration was employed. All testing procedures were automated via Python scripts, and the correctness of the implemented programs was verified using predefined test cases.

### 5.1. Experimental Results for the IEEE39-Node System

This section aims to validate the effectiveness of the proposed gravity-based algorithm to identify critical nodes. First, the composite importance score of each node is calculated based on the network topology and power flow operational data, and all nodes are ranked accordingly. The critical nodes are then removed sequentially according to the ranking, and the resulting load loss is assessed to quantify the model’s capability in identifying vulnerabilities within the power grid.

**Figure 5:**
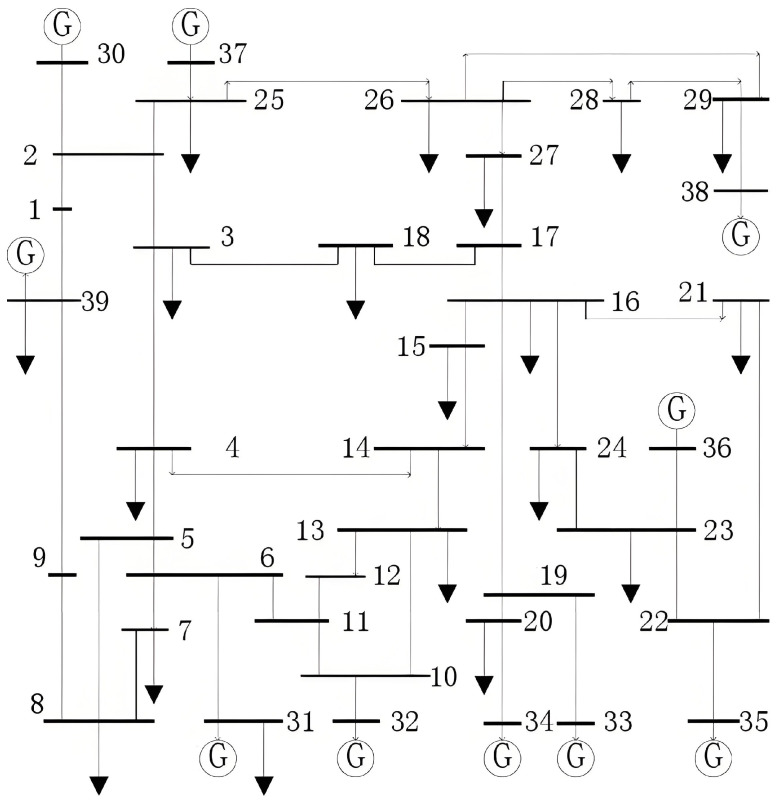
IEEE39-node system [30].

Taking the IEEE39-node system as an example, the network structure is shown in Figure 5. The IEEE 39-node system and other IEEE systems mentioned subsequently are classic benchmark test power systems that are widely used for power system analysis and algorithm validation. Originally designed by the Institute of Electrical and Electronics Engineers (IEEE), the system was intended to provide a representative medium-sized power network to test the stability, control, and optimization algorithms of power systems. The system consists of 39 nodes, including 10 generator nodes, with node 39 designated as the slack node. This network topology incorporates multi-level characteristics of generation, transmission, and distribution, making it a suitable standard test system for the validation of algorithm performance.

To validate the relative advantage of the proposed algorithm, six representative traditional methods of identifying critical nodes were selected for comparison. These methods are widely used in power network analysis and include degree centrality, the K-shell algorithm, a gravity model based on K-shell, the fused gravity model, structural holes, and electrical coupling connectivity. Table 1 summarizes the fundamental attributes of these methods. The classification is based on two dimensions: the feature domain and the scope of the analysis. The feature domain describes the type of information that each method focuses on, while the analysis scope indicates the spatial granularity at which nodes are evaluated. This classification helps to clarify the differences among the methods in terms of their ability to capture the network structure and integrate operational characteristics.

**Figure 6:**
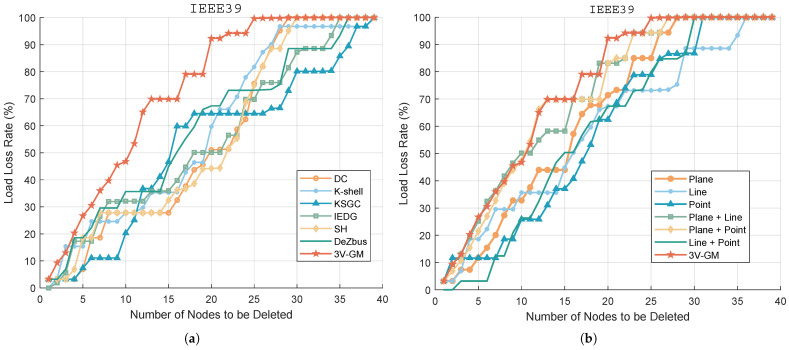
Load loss rate under an ordered node attack. (**a**) Performance comparison between the proposed 3V-GM method and six baseline algorithms (summaries of baseline methods are provided in Table 1). (**b**) Ablation study of the proposed point–line–plane framework, including plane-only importance, line-only importance, point-only importance, and the combined configurations of line + plane, line + point, and plane + point.

Figure 6a shows the impact of node removal, based on different algorithms, on the load loss rate in the IEEE 39-node system. The x-axis represents the number of removed nodes, while the y-axis indicates the load loss percentage. The experimental results show that the proposed algorithm consistently leads to a higher load loss rate compared to other methods when the same number of nodes is removed. This is particularly evident when only a few nodes are deleted, demonstrating the algorithm’s superior sensitivity in identifying structurally and electrically critical nodes. The failure of such nodes significantly reduces the system’s stability and load-carrying capacity. Although the other algorithms also exhibit an increasing trend in the load loss rate as more nodes are removed, the overall growth is more gradual, and the final load loss levels remain significantly lower than those of the proposed method. This comparison demonstrates the superiority of the proposed algorithm in localizing critical nodes and assessing network vulnerability. It offers more practical theoretical support and decision-making guidance for risk prediction, operational maintenance, and strategic protection planning in complex power networks.

To evaluate the contribution of each layer in 3V-GM, we conduct ablation experiments. Figure 6b illustrates the variation in the load loss rate during progressive node removal in the IEEE39 system for different feature configurations, including plane-only, line-only, point-only, their pairwise combinations, and the complete 3V-GM framework. It can be observed that 3V-GM achieves a higher overall load loss rate than all single-layer methods and their combinations, indicating that the integration of the three feature layers significantly improves the identification of critical nodes. The simulation results demonstrate that each feature layer in the proposed method is indispensable, and the synergistic fusion of all three layers is crucial to fully capture the structural and electrical characteristics of the power grids, thus validating the effectiveness and significance of the 3V-GM approach.

### 5.2. Experimental Results for Other IEEE Systems

**Figure 7:**
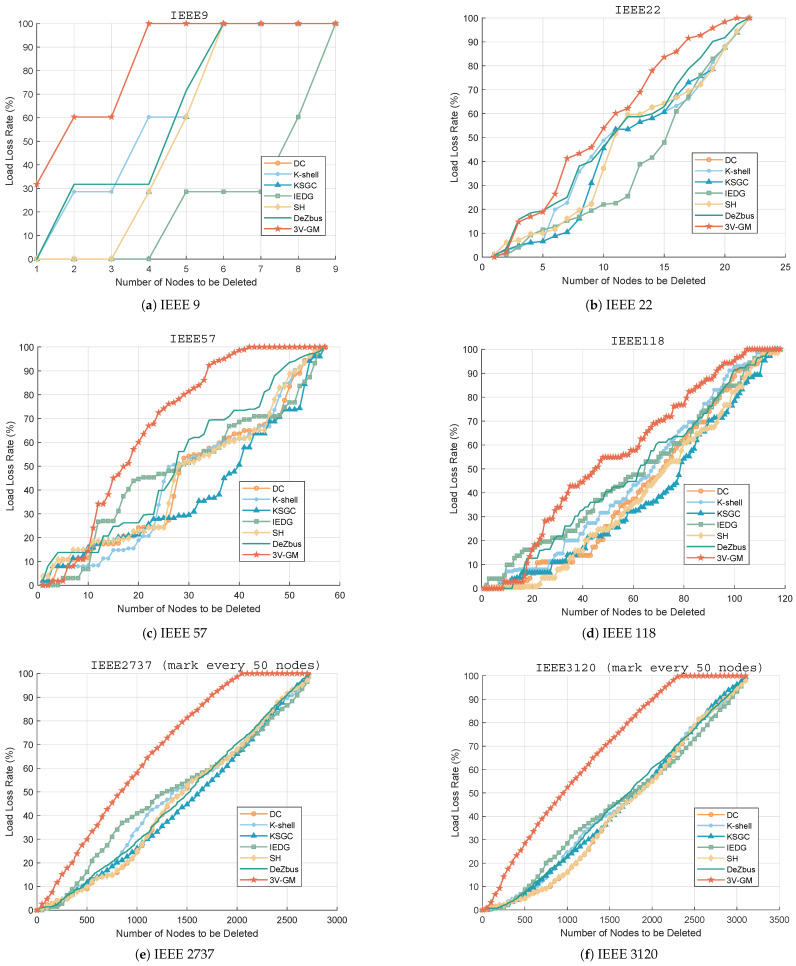
Load loss rate under ordered node attack. (**a**) IEEE 9. (**b**) IEEE 22. (**c**) IEEE 57. (**d**) IEEE 118. (**e**) IEEE 2737. (**f**) IEEE 3120.

In addition, we apply 3V-GM to classical networks of varying scales to comprehensively evaluate its effectiveness. Detailed information about the network datasets is provided in Table 2. The corresponding load loss results are illustrated in Figure 7.

As shown in Figure 7, stepwise node removal simulations were performed on multiple power networks based on criticality classification. Specifically, as illustrated in Figure 7b–d, although the initial performance of the proposed 3V-GM was not always optimal, as the number of deleted nodes increased, its load loss rate was the first to reach 1 and it almost always outperformed other methods. Furthermore, as seen in Figure 7a,e,f, the proposed method consistently maintained superior performance, demonstrating its overall advantage. Specifically, as shown in Table 3, when 20% of the nodes are removed, 3V-GM causes a 33.03% average load loss rate across all experimental networks, far exceeding those of DeZbus (19.31%), IEDC (15.87%), and K-shell (13.88%). This advantage amplifies as the removal ratio increases, reaching 97.86% at 80% removal, compared to the next-best method, DeZbus (84.34%). By accurately prioritizing high-impact areas, the method provides robust decision support for the maintenance of grid stability and operational efficiency, forming a strong basis for the optimization of scheduling strategies and implementation of targeted node protection measures.

### 5.3. Computational Example

**Figure 8:**
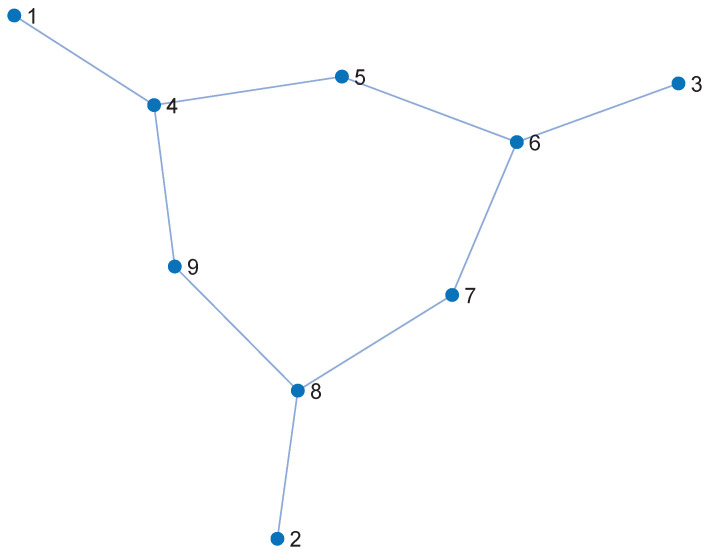
Topological structure diagram of the IEEE 9-node system.

Figure 8 illustrates the topological structure of the IEEE 9-node system, offering an intuitive view of the interconnections between nodes. Table 4 presents the importance scores for each node, as determined by various methods. This table illustrates the step-by-step computation and integration of the three feature layers in 3V-GM, serving as a reproducible computational example. Compared to traditional methods, 3V-GM delivers higher score differentiation between different nodes, avoiding the issue of nodes having the same importance score, which makes ranking difficult.

## 6. Discussion and Conclusions

With the increasing penetration of renewables and the growing complexity of modern grids, traditional methods are becoming insufficient to capture the true operational impacts of node failures. To address this limitation, we introduce 3V-GM, a “point–line–plane” framework that integrates both electrical and topological information to evaluate node criticality. The method combines the minimum-resistance surface and the expansion resistance factor at the plane level, the electrical coupling distance at the line level, and the centrality of the eigenvector at the point level. Additionally, we incorporate a dynamic load redistribution model that simulates real-world dispatch decisions, reallocating the load from failed nodes to neighboring nodes based on their capacity and treating any excess as load loss to reflect blackout risks. Our experimental results demonstrate that, in terms of the load loss rate, the 3V-GM model outperforms all six traditional algorithms used for comparison. Specifically, when 20%, 40%, 60%, and 80% of the nodes are removed, the 3V-GM method achieves the highest load loss rate (LR) across the IEEE benchmark networks. The average load losses obtained by 3V-GM are 33.03%, 66.16%, 85.45%, and 97.86%, which are significantly higher than those of the second-best method, DeZbus, i.e., 19.31%, 36.41%, 63.66%, and 84.34%, corresponding to improvements of +13.72, +29.75, +21.79, and +13.52 percentage points. As the proportion of removed nodes increases, 3V-GM is also the first to drive the LR close to or equal to 1, demonstrating that the identified nodes are critical to system stability. This enhanced performance can be largely attributed to the three-layer feature fusion strategy, which captures the structural and electrical characteristics of the grid from complementary perspectives. Further validation through ablation studies, where the layers were independently recombined, highlights the essential contribution of each layer to the overall performance of the model. In conclusion, the proposed 3V-GM algorithm demonstrates superior sensitivity to identify critical nodes and offers a more accurate assessment of system vulnerability, making it a promising tool for enhanced grid resilience. Future work will explore the integration of real-time data and artificial intelligence to further refine 3V-GM for large-scale and integrated energy systems.

## Figures and Tables

**Table 1:** Comparative attributes of representative critical node identification algorithms in power networks.

Symbol	Method	Feature Domain	Analysis Scope
DC	Degree Centrality	Topological	Local
K-Shell	K-Shell Algorithm	Topological	Global
KSGC	Improved Gravity Model	Topological	Local + Global
IEDG	Effective Distance Fusion Model	Topological	Local + Global
SH	Structural Hole	Topological	Local
DeZbus	Electrical Coupling Connectivity	Operational	Global
3V-GM	“Point–Line–Plane”	Topological + Operational	Global + Local

**Table 2:** Power network dataset.

Network	Number of Nodes	Number of Sides	Network Diameter	Average Degree	Average Path Length
IEEE9 ^1^	9	9	4	2	2.07
IEEE22 ^2^	22	21	12	1.91	4.94
IEEE39 ^3^	39	46	10	2.36	4.63
IEEE57 ^4^	57	80	12	2.74	4.87
IEEE118 ^5^	118	186	14	3.03	6.26
IEEE2737 ^6^	2737	3490	30	2.56	13.39
IEEE3120 ^7^	3120	3684	36	2.36	14.26

^1^ IEEE9: A small and highly simplified power network that, despite its limited scale, captures the fundamental characteristics of power flow and stability in real systems [31]. ^2^ IEEE22: A relatively sparse structure that mainly reflects the hierarchical and radial characteristics of distribution networks [32]. ^3^ IEEE39: (New England system) Contains 10 generator nodes with a more complex structure, representing the interconnection features of a typical regional grid [17,33]. ^4^ IEEE57: A medium-scale transmission network with more nodes and lines, suitable for reflecting the coupling between reactive power and voltage [34]. ^5^ IEEE118: Exhibits a power-law degree distribution and small-world characteristics; a few highly connected nodes play a critical role in the system structure [35]. ^6^ IEEE2737: A large-scale distribution system with a hierarchical and extensive topology, capturing the complexity of medium- and low-voltage distribution networks [36]. ^7^ IEEE3120: A large-scale network with a dense structure, reflecting the complex connectivity and scalability of huge power grids [37].

**Table 3:** Comparison of average load loss rates across node removal percentages.

Node Removal Percentage	DC	K-Shell	KSGC	IEDC	SH	DeZbus	3V-GM
20%	11.94%	13.88%	8.93%	15.87%	11.41%	19.31%	33.03%
40%	26.29%	38.24%	32.69%	31.64%	27.55%	36.41%	66.16%
60%	56.18%	58.16%	51.26%	49.62%	55.14%	63.66%	85.45%
80%	82.54%	82.03%	78.29%	70.73%	81.68%	84.34%	97.86%

**Table 4:** Importance scores for each node, calculated using various methods, including traditional approaches and the 3V-GM method, with the step-by-step integration of the feature layers.

	Node	1	2	3	4	5	6	7	8	9
Others	DC	0.33	0.33	0.33	1	0.67	1	0.67	1	0.67
	KS	1	1	1	2	2	2	2	2	2
	SH	1	1	1	3	2	3	2	3	2
3V-GM	R	0.7510	1	0.5253	0	0.2944	0	0.4126	0	0.1442
	I	0.6259	0.8333	0.4377	0	0.4906	0	0.6877	0	0.2404
	e	0.1826	0.1826	0.1826	0.4082	0.3651	0.4082	0.3651	0.4082	0.3651
	3V-GM	2.4833	3.5905	1.7579	0	4.3415	0	6.1972	0	2.7844

## Data Availability

All relevant data are available at https://github.com/D-youzi/Power-Network-Dataset-3V-GM (accessed on 6 June 2025).
